# Literary Notes

**Published:** 1899-09

**Authors:** 


### LITERARY NOTES.

---

WARNER'S Pocket Medical Dictionary is a most convenient little work. The latest medical terms have all been added, 10,400 words, terms and phrases are spelled, pronounced and defined. The definitions are concise and comprehensive and the type is bold and easily readable. The paper and binding are especially serviceable. It is bound in flexible leather, with round corners, colored edges. It contains complete tables of arteries, bacilli, spirilli, streptococci, micrococci, bacteria, muscles, nerves, and a dose table. This latter comprises a complete list of all drugs with their doses arranged in apothecaries' measure and their metric equivalents. It is well written and will prove a valuable addition to the library of quick reference books of any physician. It will be sent to any address upon receipt of 75c., stamps or money order. Address, W. R. Warner & Co., Philadelphia.

---

THE St. Louis *Courier of Medicine* which was published for more than ten years, during which time it attained high rank as a progressive medical journal, and was afterward discontinued, has been revived. The present editors are: Dr. C. R. Dudley, editor-in-chief, and Drs. Joseph Grindon, Elsworth F. Smith, W. A. Shoemaker, associate editors. We welcome the return of this sturdy journal with cordial greeting.

---

THE Texas *Clinic* is the name of a monthly journal devoted to practical medicine and surgery, published at Dallas, Texas. It is edited by Drs. P. L. Campbell, S. E. Milliken, E. A. Aronson and J. E. Wilson. The first numbers make an excellent appearance. The subscription price is $1.00 a year.

---

THE *Quarterly Medical Journal* will hereafter be edited by Christopher Addison., M.D., B.S. Lond., F.R.C.S. All letters or exchanges should in future be addressed to "The Editor, 7, Leopold Street, Sheffield."

The editor of this excellent magazine announces that it will in future be issued in the months of November, February, May and August, instead of in October, January, April and July, as has previously been the case.

THE *Occidental Medical Times*, which has for twelve years been published at Sacramento has been removed to San Francisco and consolidated with the *Pacific Record of Medicine and Surgery*. The former editor of the *Medical Times* assumes editorial management of the new magazine, with the former editor of the *Record* as his assistant. We join with the editors in anticipating in this union of interests an improvement in the medical journalism of the Coast.—*Columbus Medical Journal.*

THE Illinois *Medical Journal* has lately been issued as the official organ of the Illinois State Medical Society. It is a monthly bulletin, edited by a publication committee made up as follows: Dr. E. W. Weis, chairman, Ottawa; Dr. H. M. Moyer, Chicago, and Dr. G. N. Kreider, Springfield. The July issue contains the proceedings of the last meeting of the society at Cairo, several original articles, editorials, biographies, correspondence, news items, and the like. The arrangement is good and it is well edited.

